# Springtime photoinhibition constrains regeneration of forest floor seedlings of *Abies sachalinensis* after a removal of canopy trees during winter

**DOI:** 10.1038/s41598-018-24711-6

**Published:** 2018-04-20

**Authors:** Mitsutoshi Kitao, Hisanori Harayama, Qingmin Han, Evgenios Agathokleous, Akira Uemura, Naoyuki Furuya, Satoshi Ishibashi

**Affiliations:** 1 Hokkaido Research Center, Forestry and Forest Products Research Institute, Hitsujigaoka 7, Sapporo, 062-8516 Japan; 2 Department of Plant Ecology, Forestry and Forest Products Research Institute, Matsunosato 1, Tsukuba, 305-8687 Japan

## Abstract

A clear-cutting of canopy trees during winter often causes severe foliar damage during the following spring in forest floor seedlings of *Abies sachalinensis*, a typical shade-tolerant evergreen coniferous species. The maximum photochemical efficiency of photosystem II after an overnight dark adaptation showed a temporary decrease immediately before budbreak in 1-year-old shoots of *A. sachalinensis* seedlings grown under full sunlight in a nursery, suggesting “springtime photoinhibition” related to the phenology of evergreen coniferous species. In the field, a greater rate of canopy tree cutting during winter was associated with more severe photoinhibition in the following spring, immediately before budbreak, which subsequently resulted in a reduction in carbon gain in 1-year-old shoots, and consequently suppressed the growth of current-year shoots. Although photoinhibition under low temperature is a well-known factor to determine the survival rate of tree seedlings during winter in cool regions, the present study additionally proposes that the temporary increase in the susceptibility to photoinhibition in springtime i.e. “springtime photoinhibition” would be a constraint for the regeneration of coniferous seedlings especially when the canopy trees are removed during winter.

## Introduction

Evergreen conifers are known to develop new shoots by utilizing photosynthates recently assimilated by pre-existing shoots in spring^1–3^. Therefore, one-year-old shoots of evergreen conifers act both as a photosynthetic apparatus and as a carbohydrate pool before budbreak, as indicated by the seasonal change in leaf starch content^4–6^. Thus, pre-existing shoots of evergreen conifers experience drastic physiological changes from the recovery of winter dormancy to the onset of budbreak in spring.

*Abies sachalinensis* is a typical shade-tolerant evergreen conifer in boreal forests of northern Japan^7^. A clear-cutting of canopy trees, commonly conducted during winter, results in severe foliar damage in the shade-acclimated forest floor seedlings of *A. sachalinensis* during the following spring, sometimes leading to plant death. This suggests that an increase in the irradiance during springtime would not necessarily produce a positive effect on the growth and survival of forest floor seedlings of *A. sachalinensis*^8,9^.

Regarding the physiological changes occurring during springtime, late spring frost can cause severe damage to mature pre-existing needles of coniferous species just before the budbreak^10,11^. A temporary increase in the susceptibility to freezing temperature (−5.5 °C, artificially imposed for simulating late frost) has been reported for 1-year-old shoots of Sakhalin spruce (*Picea glehnii*), an evergreen conifer species native to northern Japan, specifically before the budbreak^10^. Similarly, Sakhalin spruce showed a significant increase in the susceptibility to photoinhibition, as indicated by a decrease in the maximum photochemical efficiency (F_v_/F_m_)^12^ in 1-year-old shoots just before the budbreak^13^.

Photoinhibition is exacerbated under low temperatures through an increase in excitation pressure and/or a suppression of photosystem (PS) II repair cycle^12,14–17^, which could be a factor determining tree growth and distribution in cool regions, including northern Japan^18–22^. Although the mechanism underlying the temporary increase in the susceptibility to photoinhibition before budbreak is unclear, it may associate with physiological changes occurring to facilitate the development of new shoots^5,13,23^. In this context, shade-adapted seedlings of *A. sachalinensis* may also suffer from photoinhibition before the budbreak during the next spring following a winter canopy tree cutting. Furthermore, shade-acclimated foliage would exhibit a greater extent of photoinhibition when exposed to full sunlight^24^.

We hypothesized that an increase in the incident irradiance by a canopy tree cutting during winter may result in photoinhibition in needles of 1-year-old shoots of *A. sachalinensis* before the budbreak in the following spring, thus suppressing the growth of current-year shoots. To test our hypothesis, we first investigated the seasonal change in F_v_/F_m_ in seedlings of *A. sachalinensis* grown in a nursery under full sunlight to clarify if the susceptibility to photoinhibition in the pre-existing 1-year-old shoots of *A. sachalinensis* increases before budbreak, as observed in Sakhalin spruce^13^. We then investigated photoinhibition in 1-year-old shoots and relative growth of current-year-shoots in seedlings of *A. sachalinensis* in a field site as a function of incident irradiance after canopy tree cuttings.

## Results

### Seasonal change in Fv/Fm in the seedlings grown in the nursery

**Figure 1:**
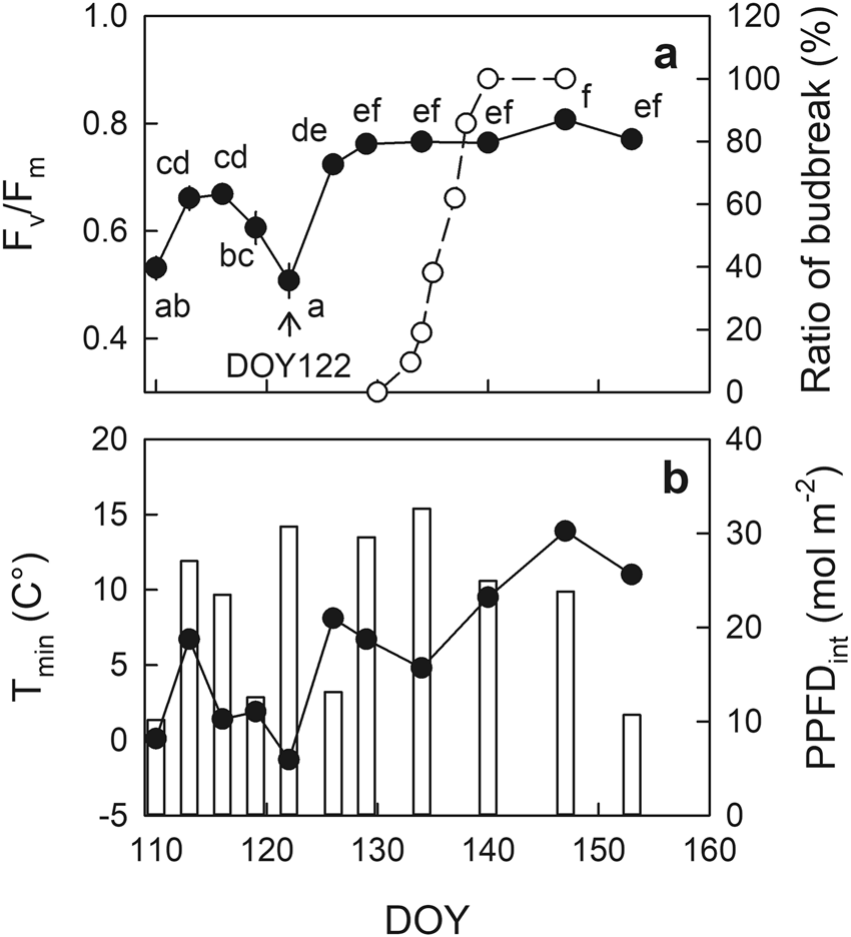
Seasonal changes in overnight dark-adapted maximum photochemical efficiency of photosystem II (F_v_/F_m_) in 1-year-old shoots in seedlings (closed circles) and the ratio of the budbreak-commenced seedlings to the total (open circles) of *Abies sachalinensis* (**a**), and minimum temperature (T_min_) (closed circles) and integrated photosynthetic photon flux density during the morning from sunrise to noon (PPFD_int_) (open bars) of the previous day to the F_v_/F_m_ measurement (**b**). Values are means ± standard error (SE) of n = 5–7 for F_v_/F_m_.

**Figure 2:**
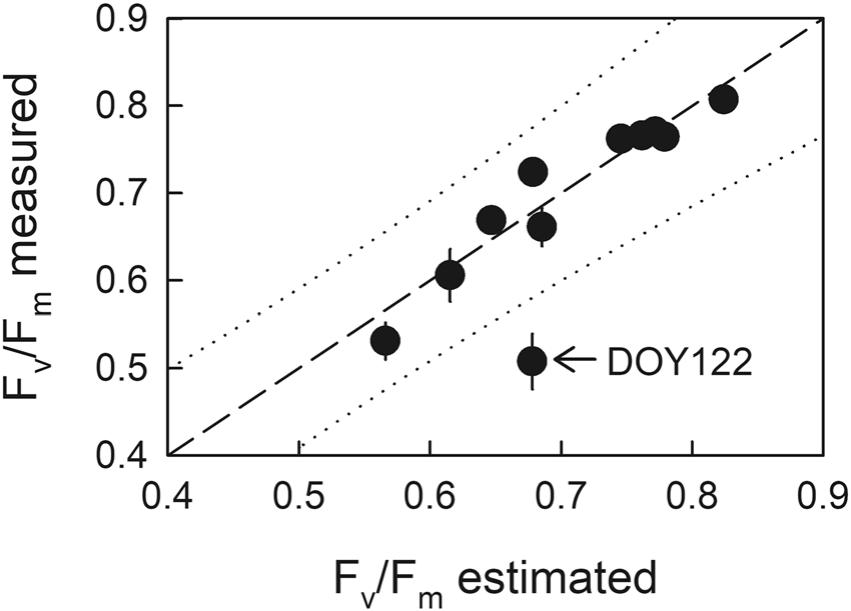
The relationship between F_v_/F_m_ measured, and F_v_/F_m_ estimated based on the multiple regression analysis. Dashed line indicates a 1:1 relationship. Dotted lines indicate the prediction interval at the level of 95%. Values are means ± standard error (SE) of n = 5–7.

In 2016, budbreak in seedlings grown in the nursery under full sunlight started from DOY 133 (May 12) and was completed by DOY 140 (May 19), as all the selected 21 seedlings had commenced budbreak in the topmost lateral shoots (Fig. 1a). Chronic photoinhibition was determined as a sustainable decrease in F_v_/F_m_ after an overnight dark-adaptation, which would be an indicator of the prolonged environmental stress^25^, especially reflecting the integrated response during the previous day. The F_v_/F_m_ after an overnight dark adaptation increased from DOY 110 to 116 (April 19 to 25), decreased to reach the minimum limit on DOY 122 (May 1), and then increased again and remained stable from DOY 129 (May 8) onwards (Fig. 1a, monofactorial ANOVA: F_10,58_ = 33.6, p < 0.001). Daily minimum air temperature (T_min_) monitored at the nursery showed the lowest value around the time that the minimum F_v_/F_m_ was observed, i.e., DOY122 (May 1) (Fig. 1b). Multiple regression analysis was conducted to investigate the effects of DOY, T_min_, and integrated PPFD during the morning from sunrise to noon (PPFD_int_) on the seasonal change in F_v_/F_m_. Multiple regression analysis, excluding DOY122, resulted in a more predictive model as F_v_/F_m_ = 0.176 + 0.00313 DOY + 0.00630 T_min_ + 0.00404 PPFD_int_ (*r*^2^ = 0.76, AIC = −210) (Table 1), compared with that including DOY122 as F_v_/F_m_ = 0.524 + 0.0191 T_min_ + 0.00254 PPFD_int_ (*r*^2^ = 0.68, AIC = −187) (Table S1). The seasonal change in F_v_/F_m_ around budbreak was well predicted by the former model, except for the apparently lower value of F_v_/F_m_ observed on DOY 122 (Fig. 2).

**Table 1:** Summary of multiple linear regression for the seasonal change in F_v_/F_m_ related to environmental factors.

Dependent variable	Summary measures			Regression coefficients			
	*r* ^2^	*P*	AIC	Independent variable	Coefficients	*P*	VIF
F_v_/F_m_	0.76	<0.001	−210	DOY	0.00313	<0.001	3.35
				T_min_	0.00630	<0.05	3.37
				PPFD_int_	0.00404	<0.001	1.01
				(Intercept)	0.176	<0.05	

Initial explanatory factors affecting F_v_/F_m_: day of year (DOY), daily minimum air temperature (T_min_), and integrated PPFD during the morning (PPFD_int_). The data of F_v_/F_m_ on May 1 (DOY 122) was excluded for the analysis as an outlier. Stepwise regressions were undertaken to define the subset of effects that would altogether provide the smallest Akaike information criterion (AIC) in subsequent modeling. As a measure of multicollinearity, variance inflation factor (VIF) is demonstrated.

### Field study

**Figure 3:**
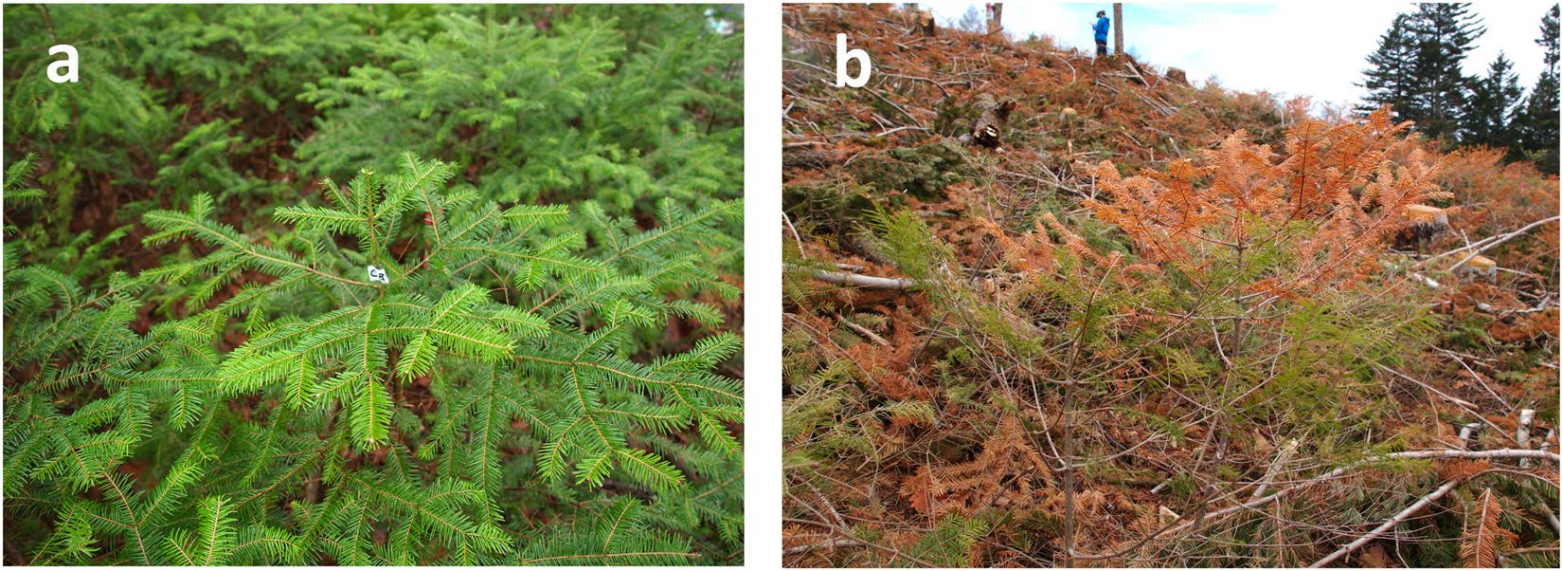
Seedlings of *Abies sachalinensis* grown either under the control canopy trees (0% canopy tree cutting) (**a**) or in the clear-cutting plot (100% canopy tree cutting), thus displaying severe foliar injury (**b**). Photographs were taken at the beginning of May, 2016.

Canopy tree cuttings were conducted at the rates of 33, 66 and 100% in October 2015 and January 2016, in the field study site of the Shibecha National Forest. Severe foliar injury was observed in seedlings grown in the plots of clear-cutting (100%) at the beginning of May 2016 (Fig. 3). Measurements of F_v_/F_m_ were conducted for 1-year-old shoots, still remaining green, in the survived seedlings. Means of average air temperature (T_avg_), T_min_ and maximum air temperature (T_max_) measured from May 11 to July 31, 2016 were not significantly different among the plots with different cutting-rates (Table S2). The arithmetic difference in the mean T_max_ among the plots was at most 2 °C, whereas there was a small difference in the mean T_min_ among the plots (within 0.2 °C). Two-way ANOVA showed no significant difference in the F_v_/F_m_ measured in May 2016 between the autumn and winter cuttings (F_1,22_ = 3.39, P = 0.08). However, the cutting-rate significantly affected F_v_/F_m_ (F_2,22_ = 52.8, P < 0.001) (Table S3). Based on the preliminary measurements, the average F_v_/F_m_ measured immediately after the autumn cuttings in October 2015 was substantially low (0.071-0.096) and statistically non-different among the four cutting rates (Table S4).

**Figure 4:**
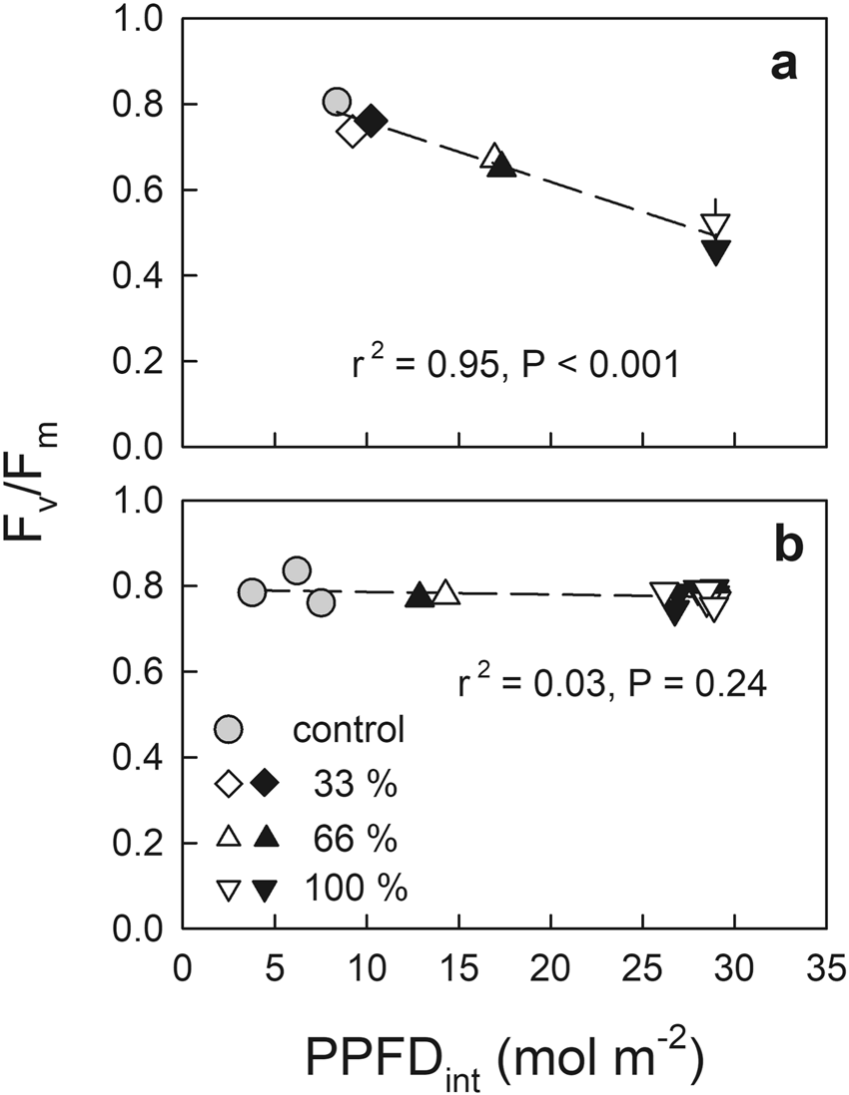
Overnight dark-adapted maximum photochemical efficiency of photosystem II (F_v_/F_m_) as a function of integrated PPFD during the morning (PPFD_int_) in 1-year-old shoots of field-grown *Abies sachalinensis* measured immediately before budbreak in springtime (**a**) and summertime (**b**), with various rates of canopy tree cutting conducted in October 2015 (open symbols) and January 2016 (closed symbols). The PPFD_int_ was estimated for May 10, 2016 (**a**) and July 25, 2016 (**b**) based on a hemispheric photograph for each plot. Values are means ± standard error (SE) of n = 3–5.

Budbreak in the field site occurred around May 20, 2016 based on the periodical photographs taken by a time lapse camera (GardenWatchCam, Brinno Solutions, WALNUT, CA, USA). The F_v_/F_m_ measurements in spring were conducted on May 11, approximately ten days before budbreak. The T_min_ during the morning of the previous day to the F_v_/F_m_ measurements was relatively moderate (5.2 °C). F_v_/F_m_ as a function of the PPFD_int_ immediately before budbreak (May 10), estimated from the hemispheric photograph, showed a decreasing trend with an increase in the PPFD_int_ (Fig. 4a). Conversely, the F_v_/F_m_ measured in summer (July 26) showed a relatively higher value of approximately 0.8 and no significant trend was observed as a function of PPFD_int_ estimated for July 25 (Fig. 4b).

**Figure 5:**
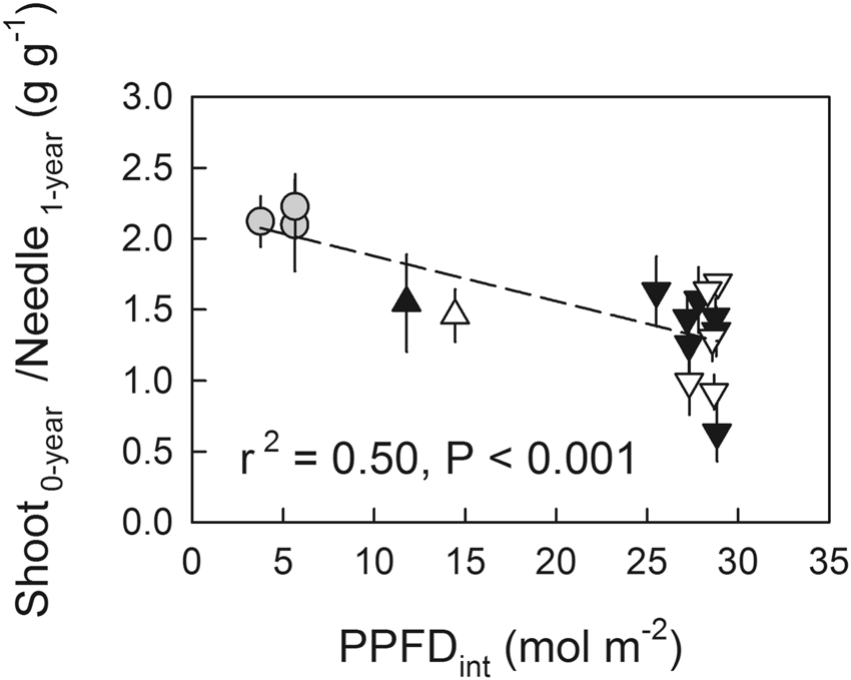
The ratio of dry weight of current-year shoots to estimated needle dry weight of 1-year-old shoot (Shoot_0-year_/Needle_1-year_) as a function of integrated PPFD during the morning from sunrise to noon (PPFD_int_), with various rates of canopy tree cutting conducted in October 2015 (open symbols) and January 2016 (closed symbols). Symbols are the same as in Fig. 4. Shoot_0-year_ includes dry weight of needle leaves and stem. The PPFD_int_ was estimated for May 10, 2016 based on a hemispheric photograph taken in July for each plot. Values are means ± standard error (SE) of n = 3–5.

As a measure of photoinhibitory damage in the 1-year-old shoots, the ratio of dry weight of current shoots to the estimated dry weight of the needles of a 1-year-old shoot (Shoot_0-year_/Needle_1-year_) was used. Across the different rates of canopy tree cutting, lower Shoot_0-year_/Needle_1-year_ was observed with a greater amount of PPFD_int_ estimated for May 10 (Fig. 5), which is closely correlated with the extent of spring photoinhibition (Fig. 4a).

## Discussion

A substantially great decline in the predawn F_v_/F_m_ was observed temporarily on DOY122 in seedlings grown under full sunlight in the nursery (≈0.67–0.5) 10 days before the onset of budbreak (Fig. 1a). Based on the multiple regression analysis, an ontogenetic recovery in photosynthetic activity from the winter dormancy was indicated by the positive coefficient for DOY (Table 1). Decreasing daily T_min_ was associated with decreasing F_v_/F_m_ (Table 1), which might be involved in “low-temperature photoinhibition”^18–22^. Although greater insolation in the morning (PPFD_int_) following low nocturnal temperature is known to exacerbate photoinhibition^18–20^, there was a positive association of PPFD_int_ with F_v_/F_m_ in the present study (Table 1). Greater PPFD_int_ might associate with accelerated ontogenetic recovery from the winter dormancy in these seedlings which were acclimated to full sunlight. Even when taking environmental factors into account, lower F_v_/F_m_ was still observed on DOY122 (Fig. 2), which supports the assertion of an intrinsic increase in susceptibility to photoinhibition immediately before budbreak i.e. “springtime photoinhibition” for *A. sachalinensis*, irrespective of environmental factors.

A temporary decline in the F_v_/F_m_ after an overnight dark adaptation immediately before budbreak has been reported for Sakhalin spruce^13^. In the previous study, air temperature gradually increased toward budbreak; however, F_v_/F_m_ in Sakhalin spruce showed a temporary decrease from 0.79 to 0.73, suggesting an increase in the susceptibility to photoinhibition before budbreak as an intrinsic phenomenon^13^. Such a temporary decrease in the F_v_/F_m_ in spring immediately before a full recovery from winter dormancy was also observed in Scots pine (*Pinus sylvestris*)^26^.

Regarding physiological changes around budbreak, an increase in the vulnerability to freezing temperature, specifically before budbreak, in 1-year-old shoots of Sakhalin spruce was also reported^10^. Rossi, Rathgeber & Deslauriers^27^ reported that onset of xylem development preceded the onset of bud break. Although further investigation is required to clarify the mechanism underlying the temporary decline in F_v_/F_m_, pre-existing shoots of *A. sachalinensis* may become more vulnerable to environmental stresses, specifically just before budbreak, which may relate to phenological changes in the entire plant. In this context, 1-year-old shoots of *A. sachalinensis* seedlings grown in the field site should also be vulnerable to photoinhibition at the beginning of May, immediately before budbreak. Lower F_v_/F_m_ was observed in the plots receiving a greater amount of insolation in the morning (Fig. 4a), although no significant trend in F_v_/F_m_ was observed during late July (Fig. 4b), suggesting a greater risk of photoinhibition in springtime for the shade-acclimated seedlings. As the *A. sachalinensis* seedlings may have already been under winter dormancy during the canopy tree cuttings in October 2015, indicated by the substantially low F_v_/F_m_ (Table S4)^28,29^, they equally suffered photoinhibition during the following spring irrespective of the timing of canopy tree cutting (Table S3, Fig. 4).

A considerable number of needles were detached from the damaged 1-year-old shoots by the end of July. As photosynthesis in the current-year shoot might not have been fully activated by the end of July^6,30^, the growth of current-year shoots measured in July was considered to mainly reflect the amount of photosynthetic carbon gain in the 1-year-old shoots. Because the extents of photoinhibition measured in spring were greater in the seedlings receiving greater amounts of irradiance in the morning (Fig. 4a), smaller Shoot_0-year_/Needle_1-year_ with a greater amount of irradiance in May suggests that photoinhibitory damage in springtime resulted in a smaller amount of carbon gain in 1-year-old shoots because of suppressed photosynthetic rate^31,32^ and leaf defoliation.

Winter desiccation occasionally occurs in trees grown in the eastern part of Hokkaido because of water unavailability when soil is frozen in winter^33^. Based on the measurement of water potential and staining of conductive xylem in seedlings grown in the field site, which were sampled at the beginning of May, stem hydraulic conductivity was maintained, even in the seedlings with severe foliar damage (Harayama, unpublished data). Thus, photoinhibitory damage because of the increased irradiance might be a major factor determining the growth and survival of seedlings of *A. sachalinensis* after canopy tree cutting. It should be noted that lower leaf temperature due to nighttime radiational cooling in sky-exposed seedlings after canopy tree cutting could be an additional contributor to low-temperature photoinhibition before bud break, compared with seedlings grown in a forest understory^18–20^.

In conclusion, the greater rate of canopy tree cutting during winter was associated with more severe photoinhibition during the following spring, possibly immediately before budbreak, which subsequently resulted in a reduction in carbon gain in the 1-year-old shoots, and consequently suppressed the growth of current-year shoots. Although photoinhibition under low temperature during winter is an important factor to determine the survival rate of tree seedlings in cool regions^18–22^, the present study additionally proposes that “springtime photoinhibition”, i.e. a temporary increase in the susceptibility to photoinhibition in springtime related to phenological events of evergreen coniferous species, would be a constraint for the growth and regeneration of *A. sachalinensis*. A forest logging technique that maintains a residual canopy as “sheltering trees”^18,20,34^ would enhance the regeneration of evergreen coniferous species. Furthermore, a canopy tree cutting after the decline in F_v_/F_m_ observed before budbreak would be an alternative method to alleviate the extent of photoinhibition in shade-acclimated seedlings, thereby promoting natural regeneration of evergreen coniferous species.

## Methods

### Seasonal change in the susceptibility to photoinhibition in seedlings grown under full sunlight in a nursery

Chronic photoinhibition, determined as a sustainable decrease in the maximum photochemical efficiency of photosystem (PS) II (F_v_/F_m_) after an overnight dark adaption^25,35^, was measured from the end of April 2016 to the beginning of June 2016 (around budbreak). Overnight dark-adapted F_v_/F_m_ was measured on DOY X, Y, Z to indicate the physiological state of the previous DOY (X-1, Y-1, Z-1). Seven-year-old *A. sachalinensis* seedlings (height ≈ 70 cm) were grown under full sunlight in the nursery of the Hokkaido Research Center, Forestry and Forest Products Research Institute, Sapporo, Japan [42.99°N, 141.39°E; 180 m above sea level (a.s.l.)]. Early in the evening (≈17:00) of the previous day of measurements, 1-year-old shoots in the topmost lateral shoots of five to seven *A. sachalinensis* seedlings were cut under water in the field and transported to a laboratory, ensuring that the cut edges were placed in water for overnight dark adaptation. F_v_/F_m_ was determined after an overnight dark-adaptation using a fluorometer (mini-PAM, Walz, Effeltrich, Germany), by applying a saturating light pulse at approximately 6000 µmol m^−2^ s^−1^ PFD (photon flux density) for 1 s. Twenty-one seedlings in total were randomly selected for monitoring budbreak in the topmost lateral shoots. Air temperature at the nursery was monitored at a height of 1.5 m (1-h interval). Photosynthetic photon flux density (PPFD) under full sunlight was monitored using a quantum sensor (Li-190A, Li-Cor, Lincoln, NE, USA) at the top of a flux observation tower (41.3 m) installed in the experimental forest of the Hokkaido Research Center^36^, 1 km away from the nursery.

### Field study site

A study site was established in a 62-year-old forest plantation of *A. sachalinensis* located at Shibecha, Hokkaido, Japan (43.25°N, 144.59°E, 90 m a.s.l.), 260 km eastward of Sapporo city. This plantation is a national forest managed by the Konsen Seibu regional forest office of the Forestry Agency, Japan. According to the meteorological observation data collected at Shibecha Town, the mean annual temperature and precipitation are 5.2 °C and 1,034 mm (1981–2010), respectively, and the mean maximum snow depth is 64 cm (1987–2010) (Japan Meteorological Agency, http://www.data.jma.go.jp/obd/stats/etrn/view/nml_amd_ym.php?prec_no=19&block_no=0093&year=&month=&day=&view=). In Hokkaido, the northernmost island of Japan, a considerable number of *A. sachalinensis* seedlings can be found under the canopy of adult *A. sachalinensis* trees in forest plantations. At the study site, the last thinning operation (row thinning at the rate of 25.0%) was conducted in 1995 when the stand was 43 years old. Most of the seedlings appeared and started growing during the thinning in 1995. The height of the seedlings exceeded 100 cm at the highest point. The growth of seedlings has been decreased in recent years after the canopy closure. In such a condition, canopy tree cuttings were conducted at various rates (100%, 66%, 33%, and 0% as a control) during October 2015 and January 2016. Air temperature was observed with a 2-h interval for monitoring the local conditions after cutting (after 29 October 2015 for autumn-cutting plots and control, and after 11 May 2016 for winter-cutting plots) by using temperature data loggers (HOBO pro v2, Onset computer Corporation, Bourne, MA, US).

The F_v_/F_m_ was measured after an over-night dark-adaption in foliage of 1-year-old shoots grown under various rates of canopy tree cutting, as described in the nursery study. Three to five neighboring seedlings were randomly selected as representatives for each cutting treatment. One-year-old shoots, which still remained green in the survived seedlings, were selected for the F_v_/F_m_ measurements (cf. Fig. 3). Measurements were conducted on May 11, 2016 (spring) and on July 26, 2016 (summer). At the end of July, several plots consisting of three to five seedlings within the clear-cutting sites (100% cutting rate) were additionally selected. Preliminary measurements of the F_v_/F_m_ were also conducted in 1-year-old shoots during the evening after a 15-min adaptation to the dark immediately after the canopy tree cutting in October 2015.

Light environment for each plot consisting of three to five seedlings was evaluated using hemispheric photographs, which were taken by a digital camera (Coolpix 900, Nikon, Tokyo, Japan) combined with a fisheye lens (Fisheye Lens, FC-E8, Nikon) above the seedlings. Based on the photographs, the light environment where the seedlings were grown was estimated using a canopy analysis software (WinScanopy, Regent Instruments Inc., Quebec, Canada^37^). As the combination of low nocturnal temperatures and high insolation, particularly in the morning, is considered to be critical for the survival of seedlings^14,19^, an integrated amount of solar radiation from sunrise to noon of May 10, 2016 and July 25, 2016 (days before F_v_/F_m_ measurements) was estimated using the same canopy analysis software, assuming that clouds did not interfere with solar radiation.

At the end of July, a 1-year-old shoot attached with current-year shoots was sampled for each seedling used for the measurements of chlorophyll fluorescence. The numbers of remaining needles in the 1-year-old shoot were quite different among seedlings because of a defoliation of damaged needles. There was a close positive relationship between the shoot length of 1-year-old shoots and the total dry weight of needle leaves of 1-year-old shoots in the seedlings grown under shade (control, integrated PPFD <8 mol m^−2^) without any foliar damage in July (Fig. S1). It was assumed that the estimated dry weight of needles from the shoot length of 1-year-old shoots reflected the amount of attached needles before the canopy tree cutting. The ratio of dry weight of current-year shoots to estimated dry weight of needles of a 1-year-old shoot (Shoot_0-year_/Needle_1-year_) was used to assess the potential effects of photoinhibitory damage on the carbon gain of 1-year-shoots, including a reduction in photosynthesis and defoliation.

### Statistical analysis

The effects of the date on F_v_/F_m_ in the seedlings grown in the nursery were analyzed by monofactorial ANOVA^38^. Differences in the F_v_/F_m_ among the dates were analyzed by the Holm post-hoc test. Multiple regression analysis was used for a quantitative evaluation of the influence of day of year (DOY), daily T_min_, and integrated PPFD in the morning (PPFD_int_) as the major explanatory factors on the seasonal change in F_v_/F_m_ in the nursery-grown seedlings. Stepwise regressions were undertaken to define the subset of effects that would altogether provide the smallest Akaike information criterion (AIC) in subsequent modeling. Furthermore, variance inflation factor (VIF) was also calculated as a measure of multicollinearity. It was considered that VIF > 5 constitutes a multicollinearity problem. In the field study, the effects of plots with different cutting rates on means of daily T_avg_, T_min_ and T_max_ measured from May 11 to July 31, 2016, were analyzed by monofactorial ANOVA. The effects of timing and rate of canopy tree cutting on the F_v_/F_m_ measured in May in the seedlings grown in the field were analyzed by two-way ANOVA. The results were considered significant at an α level of 0.05.

### Data availability

All data used in this manuscript are present in the manuscript and its supplementary information.

## Electronic supplementary material

Supplementary Information
