# Supplementary material for: Springtime photoinhibition constrains regeneration of forest floor seedlings of Abies sachalinensis after a removal of canopy trees during winter
Source: Sci Rep. 2018 Apr 20;8:6310. doi: 10.1038/s41598-018-24711-6 (PMC5910419; doi:10.1038/s41598-018-24711-6)

Springtime photoinhibition constrains regeneration of forest floor seedlings of *Abies sachalinensis* after a removal of canopy trees during winter

Mitsutoshi Kitao, Hisanori Harayama, Qingmin Han, Evgenios Agathokleous, Akira Uemura, Naoyuki Furuya & Satoshi Ishibashi

Table S1. Summary of multiple linear regression for the seasonal change in F_v/F_m related to environmental factors. Initial explanatory factors affecting F_v/F_m: day of year (DOY), daily minimum air temperature (T_{min}), and integrated PPFD in the morning ($PPFD_{int}$). The data of F_v/F_m on May 1 (DOY 122) was also included for the analysis (cf. Table 1). Stepwise regressions were undertaken to define the subset of effects that would altogether provide the smallest Akaike information criterion (AIC) in subsequent modeling. As a measure of multicollinearity, variance inflation factor (VIF) is demonstrated.

Dependent variable	Summary measures			Regression coefficients			
	r^2	*P*	AIC	Independent variable	Coefficients	*P*	VIF
F_v/F_m	0.68	<0.001	-187	T_{min}	0.0191	<0.001	1.02
				$PPFD_{int}$	0.00254	<0.01	1.02
				(Intercept)	0.524	<0.001	

Table S2. Means of daily average (T_{avg}), minimum (T_{min}) and maximum air temperature (T_{max}) (°C), measured from May 11 to July 31, 2016, at the field plots with various rates (0, 33, 66 and 100%) of canopy tree cutting conducted in October 2015 (autumn, A) and January 2016 (winter, W). Values are mean ± SE (n = 82). A summary of the monofactorial ANOVA to test the effects of plots with different combinations of cutting rate and timing is shown. ns indicates non-significant.

	0%	33%		66%		100%		ANOVA
		A	W	A	W	A	W	
T_{avg}	13.2 ±0.4	13.5 ±0.4	13.5 ±0.4	13.8 ±0.4	13.6 ±0.4	13.8 ±0.4	13.7 ±0.4	ns
T_{min}	9.7 ±0.5	9.7 ±0.5	9.6 ±0.5	9.6 ±0.5	9.5 ±0.5	9.5 ±0.5	9.5 ±0.5	ns
T_{max}	17.3 ±0.5	18.0 ±0.5	18.2 ±0.5	18.9 ±0.6	18.5 ±0.6	19.3 ±0.6	18.8 ±0.6	ns

Table S3. Overnight dark-adapted maximum photochemical efficiency of photosystem II (F_v/F_m) in 1-year-old shoots of field-grown *Abies sachalinensis* measured immediately before budbreak in springtime with various rates of canopy tree cutting (100%, 66%, and 33%) conducted in October 2015 (autumn, A) and January 2016 (winter, W). A summary of the two-way ANOVA to test the effects of timing (autumn, A or winter, W), cutting rate and their interaction of canopy tree cutting on F_v/F_m is shown. Values are mean ± SE (n = 3 – 5). ns indicates non-significant.

	33%		66%		100%		ANOVA		
	A	W	A	W	A	W	Timing	Rate	Interaction
F_v/F_m	0.74 ±0.02	0.76 ±0.04	0.67 ±0.03	0.65 ±0.03	0.52 ±0.09	0.46 ±0.08	ns	$P < 0.001$	ns

Table S4. Maximum photochemical efficiency of photosystem II (F_v/F_m) in 1-year-old shoots of field-grown *Abies sachalinensis* measured immediately after the autumn canopy tree cutting. A summary of the monofactorial ANOVA to test the effect of cutting rate on F_v/F_m is shown. Values are mean ± SE (n = 5). ns indicates non-significant.

	0%	33%	66%	100%	ANOVA
F_v/F_m	0.096 ±0.008	0.086 ±0.008	0.071 ±0.008	0.075 ±0.02	ns

Figure S1. The relationship between shoot length (Length $_{1\text{-year}}$) and dry weight of needle leaves (Needle $_{1\text{-year}}$) in 1-year-old shoots in the seedlings of *Abies sachalinensis* grown under shade [integrated photosynthetic photon flux density (PPFD) during the morning < 8 mol m^{-2}] without any foliar damage.

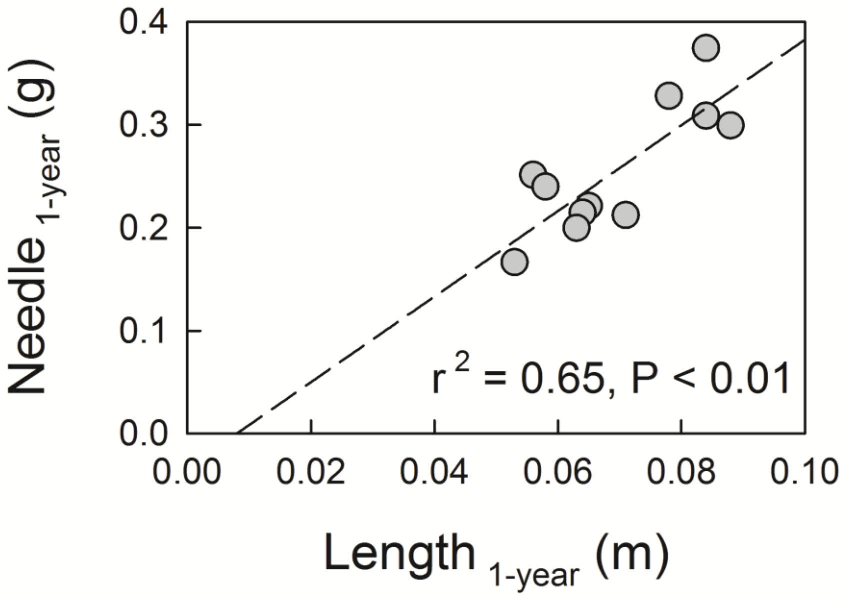

Supplement: Supplementary file 1 — Supplementary Information [file 41598_2018_24711_MOESM1_ESM.pdf]
